# The Role of Noncommunicable Disease Clinics in Improving Control of Hypertension and Diabetes Among Adults Residing in Rural Ballabgarh, Haryana

**DOI:** 10.7759/cureus.37283

**Published:** 2023-04-08

**Authors:** Aswani K Seth, Subham Kansal, Harshal R Salve, Surbhi Gupta, Rakesh Kumar, Puneet Misra

**Affiliations:** 1 Centre for Community Medicine, All India Institute of Medical Sciences, New Delhi, IND

**Keywords:** primary health centre, noncommunicable disease clinic, cvd risk score, control rates, adequate follow-up

## Abstract

Introduction

High systolic blood pressure (SBP) and raised plasma glucose are major attributable and preventable causes of death worldwide. The objective of this study was to estimate the control rates and identify determinants of control of hypertension and diabetes among adults.

Methods

A longitudinal follow-up study was conducted among all the adults registered at the noncommunicable disease (NCD) clinics under the national program at two primary health centers in Faridabad, Haryana. Data were collected every month from the individual booklet generated for registered adults. Two monthly visits in three months and four in six months were considered adequate follow-ups at the NCD clinic.

Results

In the study, 495 (82.2%) adults had hypertension, and 242 (40.2%) had diabetes. The control rates at the third and sixth months were 37.1% (95% confidence interval (CI): 31.4-42.7) and 53.6% (95% CI: 43.4-59.8) among hypertensives and 28.7% (95% CI: 21.7-35.7) and 35.9% (95% CI: 27.5-44.4) among diabetics. Among hypertensives, six-month control status was associated with adequate follow-up at the NCD clinic (adjusted odds ratio (AOR) 2.3; 95% CI: 1.4-4.0; p-value: 0.002), male sex (AOR 0.5; 95% CI: 0.3-0.9; p-value: 0.02) and high SBP (AOR 0.5; 95% CI: 0.3-0.9; p-value: 0.017).

Conclusions

Control status was achieved in half of the adults with hypertension and one-third of adults with diabetes after six months of regular follow-up. Adequate follow-up at the NCD clinic, male sex, and raised SBP emerged as determinants of control among hypertensives.

## Introduction

The burden of noncommunicable diseases (NCDs) is increasing progressively among adults in India [1]. High systolic blood pressure (SBP) and fasting plasma glucose are among the major factors responsible for global attributable deaths [2] and are two preventable causes of premature death among adults. Death can be prevented when blood pressure (BP) and plasma glucose levels are within a certain limit defined as the 'controlled' status [3,4].

The controlled status of hypertension is defined as BP < 140/90 mm of Hg and BP < 130/80 among patients with diabetes as a co-morbidity, whereas postprandial blood sugar (PPBS) less than 140 mg/dl or fasting blood sugar (FBS) less than 126 mg/dl is defined as control among patients with diabetes [5].

Uncontrolled hypertension leads to an increased risk of cardiovascular disease and stroke; on the other hand, uncontrolled diabetes leads to complications like neuropathy, nephropathy, and retinopathy [6-9]. Uncontrolled status for a longer duration leads to severe complications like non-healing ulcers, amputation, renal failure, blindness, etc. [10]. Thus, more emphasis is needed from clinicians as well policy makers on achieving target control of hypertension and diabetes status among adults [11].

The National Program for Prevention and Control of Cancer, Diabetes, Cardiovascular Diseases and Stroke (NPCDCS) was initiated in the year 2010 with a focus on enabling opportunistic screening for NCDs [5]. Opportunistic screening under the program denotes periodic screening of adults more than equal to 30 years of age for NCDs during their contact with the health facility. Opportunistic screening at all levels of a health facility will help in early detection, leading to timely initiation of treatment and ultimately controlling these chronic diseases to prevent unwanted complications [12].

NCD clinic under the NPCDCS is operational from and above the community health center level [5]. This affects the early diagnosis and initiation of treatment among adults with poor accessibility to these centers. Hypertension and diabetes are both chronic diseases and need regular care. Even though studies on control rates among adults with hypertension and diabetes in India have been published earlier, there is a dearth of published evidence on determinants of control status and effects of adequate follow-up among these adults in India and, moreover, from rural north India. Hence the objective of the study is to estimate the control rates and identify determinants of control of hypertension and diabetes among adults at third and sixth-month follow-up.

## Materials and methods

A longitudinal follow-up study was conducted among adults with hypertension and diabetes registered at the NCD clinic of primary health centers (PHCs) at Dayalpur and Chhainsa situated in the Faridabad district, Haryana, India. These PHCs cater to 28 villages with an approximate population of 1.1 lakhs and are under the rural intensive field practice area (IFPA) of All India Institute of Medical Sciences (AIIMS), New Delhi. An NCD clinic is conducted at both the PHCs on all working days, i.e., from Monday to Saturday. On average, 50-60 adults turn up at the NCD clinic daily.

Study participants were adults aged 30 years or above registered at the NCD clinic under the NPCDCS program from 1st September 2021 to 31st December 2021. All adults registered during this period were included in the study. Adults with missing baseline data on BP and FBS /random blood sugar (RBS) levels and adults with complications due to hypertension and diabetes were excluded.

Eligible adults attending the outpatient department (OPD) of both the PHCs were screened for hypertension and diabetes and the associated risk factors by trained health professionals. BP was measured after five minutes of rest using a digital sphygmomanometer. If raised, the second reading was taken after 15 minutes. Random blood sugar (capillary) level was measured using glucometers. Screened positive adults were evaluated by the medical officers at PHC; those diagnosed were registered at the NCD clinic and managed accordingly. Management of the registered adults was as per NPCDCS guidelines 2017. Month-long medication was provided to all registered adults. Adults with complications were referred to a higher center. An NCD booklet was generated for adults registered at the clinic.

Data for both baseline and follow-up were collected using the NCD booklet provided for individual adults under the NPCDCS program. Data in the booklet were filled by the consulting medical officer at follow-up visits. Baseline data included age, sex, body mass index (BMI), baseline BP and FBS/RBS, family history of the disease, history of smoking in the last year, history of alcohol intake in the last month, and WHO non-laboratory cardiovascular disease (CVD)-risk score. WHO non-laboratory CVD-risk score uses age, sex, smoking status, SBP, and BMI and generates a CVD risk score. Risk stratification is as follows <5% (very low), 5-10% (low), 10-20% (intermediate/moderate), and >20% (high). This is useful in setting with poor availability of laboratory services. A study conducted in North India showed good agreement between the WHO laboratory and non-laboratory CVD risk score [13]. BP and FBS/RBS were measured monthly at each follow-up visit. Data at the third month and sixth month of follow-up were considered to obtain control rates. A grace period of +15 days was taken for the third and sixth month data collection. Two monthly visits in three months and four in six months were considered adequate follow-ups at the NCD clinic.

STATA 17.0 (StataCorp LLC, College Station, Texas) was used for analysis. A descriptive analysis of the baseline data was done. The chi-square test of significance was applied to identify the difference between characteristics of follow-up and loss to follow-up adults. McNemar test was used to identify a significant change in control rates from baseline to three- and six-month follow-up. Determinants of control status were identified using multivariable logistic regression. Variables with a p-value of less than 0.25 in bi-variable regression were included in the adjusted model.

## Results

A total of 654 adults were registered in the NCD clinic during the study period. Around 602 (92.0%) adults were eligible for the study, and 52 (8.0%) adults were excluded due to missing baseline data. Out of all eligible adults, 495 (82.2%) had hypertension, 242 (40.2%) had diabetes, and 135 (22.4%) had both hypertension and diabetes. At three months, 286 (57.8%) adults with hypertension and 164 (67.8%) adults with diabetes were followed up. Similarly, at six months, 252 (50.9%) adults with hypertension and 128 (52.9%) adults with diabetes were followed up. There was no significant difference between the characteristics of adults who followed up and adults who were lost to follow-up.

The majority (60.6%) of the adults were female, the mean age of adults was 57.7 ± 11.8 years, and the median duration of NCD was three years with an inter-quartile range of one to five. Among all adults, 54.3% were overweight or obese (i.e., BMI >24.9 kg/m2) with a mean BMI of 25.6 ± 4.5 kg/m^2^, and 17.1% had a family history of hypertension or diabetes. The mean SBP and diastolic blood pressure (DBP) among adults with hypertension were 147.13 ± 18.7 and 94.38 ± 15.1 mm of Hg, respectively. The mean RBS among adults with diabetes was 254.5 ± 64.9 mg/dl. Median WHO non-lab CVD scores were 10 and 8.3 among adults with hypertension and diabetes, respectively (Table1).

**Table 1:** Baseline characteristics of hypertensive and diabetic patients registered at the NCD clinic HTN - hypertension, DM - diabetes mellitus, CVD - cardiovascular disease, NCD - noncommunicable disease

Variable	Category	Patients with hypertension (n= 495)	Patients with diabetes (n=242)
		Frequency (%)	Frequency (%)
Sex	Female	300 (60.6)	137 (56.6)
	Male	195 (39.4)	105 (43.4)
Age (years)	<60	284 (57.4)	151 (62.4)
	>60	211 (42.6)	91 (37.6)
Duration of disease (years)	< 3	219 (63.1)	121 (66.1)
	>3	128 (36.9)	62 (33.9)
Body mass index (Kg/m^2^)	<18.5	22 (4.4)	8 (3.3)
	18.5-24.9	198 (40.0)	105 (43.4)
	25-29.9	183 (37.0)	90 (37.2)
	>29.9	92 (18.6)	39 (16.1)
Tobacco use	Present	122 (24.7)	61 (25.2)
Alcohol consumption	Present	54 (10.9)	21 (8.7)
Family history of HTN and DM	Present	78 (15.8)	41 (16.9)
Non-lab CVD risk	Very low (< 6%)	0	83 (34.3)
	Low (<11%)	288 (58.2)	83 (34.3)
	Moderate (<21%)	181 (36.6)	70 (29.9)
	High (>21%)	26 (5.2)	6 (2.5)
Systolic blood pressure (mmHg)	<120	38 (7.7)	55 (22.7)
	120-139	120 (24.2)	91 (37.6)
	140-159	211 (42.6)	65 (26.9)
	>159	126 (25.5)	31 (12.8)
Diastolic blood pressure (mmHg)	<80	72 (14.6)	61 (25.2)
	80-89	119 (24.0)	83 (34.3)
	90-99	144 (29.1)	53 (21.9)
	>99	160 (32.3)	45 (18.6)
Random blood sugar (mg/dl)	<160	354 (71.5)	55 (22.7)
	160-219	59 (11.9)	49 (20.3)
	>219	82 (16.6)	138 (57.0)

**Figure 1:**
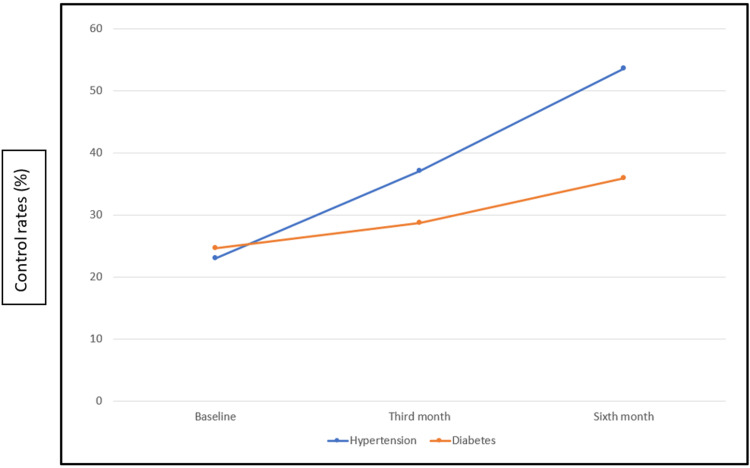
Impact of follow-up at the NCD clinic among adults with hypertension and diabetes NCD - noncommunicable disease

Control rates at the third and sixth month among adults with hypertension were 37.1% (95% CI: 31.4-42.7) and 53.6% (95% CI: 43.4-59.8), and among adults with diabetes, they were 28.7% (95% CI: 21.7-35.7) and 35.9% (95% CI: 27.5-44.4). A statistically significant improvement was observed in control rates among adults with hypertension from baseline to third month (p-value: 0.004), from baseline to sixth month (p-value: <0.001), and even from third to sixth month (p-value: <0.001). There was an improvement in control rates among adults with diabetes from baseline to third month (p-value: 0.889), from baseline to sixth month (p-value: 0.169), and from third to sixth month (p-value: 0.435) (Figure 1).

At six months follow up, among adults with hypertension, male sex (AOR- 0.5, 95% CI: 0.3-0.9; p-value: 0.02) and higher SBP (AOR-0.5, 95% CI: 0.3-0.9; p-value: 0.017) were significantly associated with control status. Similarly, there was a significant association between adequate follow-up at the NCD clinic and control status (AOR-2.3, 95% CI: 1.4-4.0; p-value: 0.002) among adults with hypertension at six months follow-up (Table 2). Among adults with diabetes, both at the third and sixth month follow-up, there is a significant negative association between higher RBS (>220mg/dl) with control status (Table 3).

**Table 2:** Determinants of control status among adults with hypertension Variables included in the adjusted model were sex, duration of disease, baseline SBP and DBP for the three-month follow-up, and sex, baseline BMI, baseline SBP and adequacy of follow-up for the six-month follow-up as p-value in the bivariable analysis was <0.250 BMI - body mass index, SBP - systolic blood pressure, DBP - diastolic blood pressure

Variable	Category	3 month follow-up (n=286)			6 month follow-up (n=252)		
		Controlled	Crude OR (95% CI)	Adjusted OR (95% CI)	Controlled	Crude OR (95% CI)	Adjusted OR (95% CI)
Age (years)	>60	57 (39.9)	1.3 (0.8-2.1)	-	71 (56.4)	1.3 (0.8-2.1)	-
Sex	Male	35 (31.8)	0.7 (0.4-1.1)	0.7 (0.4-1.1)	43 (44.3)	0.6 (0.3-0.9)	0.5 (0.3-0.9)
Duration of disease (years)	>3	45 (42.1)	1.5 (0.9-2.6)	1.4 (0.9-2.4)	48 (49.0)	0.8 (0.5-1.4)	-
Baseline BMI (Kg/m^2^)	> 25	55 (35.7)	0.9 (0.5-1.4)	-	69 (50.0)	0.7 (0.4-1.2)	0.8 (0.5-1.3)
Smoking	Present	23 (32.9)	0.8 (0.4-1.4)	-	30 (51.7)	0.9 (0.5-1.6)	-
Alcohol intake	Present	8 (29.6)	0.7 (0.3-1.6)	-	9 (36.0)	0.5 (0.2-1.1)	-
Family history	Present	17 (43.6)	1.4 (0.7-2.7)	-	20 (57.1)	1.2 (0.6-2.4)	-
Baseline SBP (mmHg)	> 140	61 (33.2)	0.6 (0.4-1.0)	0.8 (0.5-1.4)	75 (46.9)	0.5 (0.3-0.8)	0.5 (0.3-0.9)
Baseline DBP (mmHg)	> 90	51 (33.2)	0.7 (0.4-1.1)	0.8 (0.4-1.4)	66 (48.5)	0.6 (0.4-1.1)	-
Adequacy of follow up	Adequate	64 (39.5)	1.3 (0.8-2.1)	-	72 (64.3)	2.2 (1.3-3.7)	2.3 (1.4-4.0)

**Table 3:** Determinants of control status among adults with diabetes Variables included in the adjusted model were age and baseline RBS for the three-month follow-up and age, sex, duration of disease, baseline BMI and baseline RBS for the six-month follow-up as the p-value in the bivariable analysis was <0.250. BMI - body mass index, RBS - random blood sugar

Variable	Category	3 month follow up (n=286)			6 month follow up (n=252)		
		Controlled	Crude OR (95% CI)	Adjusted OR (95% CI)	Controlled	Crude OR (95% CI)	Adjusted OR (95% CI)
Age (years)	>60	23 (33.8)	1.5 (0.8-3.0)	0.9 (0.4-2.3)	25 (44.6)	2.0 (0.9-4.1)	1.4 (0.5-3.7)
Sex	Male	17 (25.0)	0.7 (0.4-1.5)	-	15 (28.3)	0.6 (0.3-1.2)	0.6 (0.3-1.5)
Duration of disease (years)	>3	16 (29.6)	1.1 (0.5- 2.3)	-	18 (42.9)	1.9 (0.9-4.1)	1.4 (0.6-3.3)
Baseline BMI (kg/m^2^)	> 25	25 (29.1)	1.0 (0.5-2.1)	-	21 (30.4)	0.6 (0.3-1.2)	0.6 (0.3-1.5)
Smoking	Yes	14 (33.3)	1.4 (0.6-2.9)	-	12 (35.3)	0.9 (0.4-2.2)	-
Alcohol intake	Yes	4 (30.8)	1.1 (0.3- 3.8)	-	2 (20.0)	0.4 (0.1-2.1)	-
Family history	Yes	7 (25.00)	0.8 (0.3-2.0)	-	9 (47.4)	1.8 (0.7-4.7)	-
Baseline RBS (mg/dl)	160-219	14 (34.2)	0.4 (0.2-1.0)	0.4 (0.2-1.0)	14 (41.2)	0.4 (0.1-1.0)	0.4 (0.1-1.0)
	>219	12 (14.1)	0.1 (0.1-0.3)	0.1 (0.1-0.3)	12 (18.8)	0.1 (0.1-0.3)	0.1 (0.1-0.4)
Adequacy of follow up	Adequate	25 (30.9)	1.2 (0.6-2.4)	-	23 (39.0)	1.3 (0.6-2.8)	-

## Discussion

The study was conducted to estimate the control rates among adults attending NCD clinics at PHCs and to determine the determinants of control among these adults.

In the present study, control rates among adults with hypertension at baseline, third- and sixth-month follow-up were 23%, 37.1%, and 53.6%, respectively. The National Noncommunicable Disease Monitoring Survey (NNMS) 2017-18 in India [14] reported control in 14.6% of adults above 30 years of age. The higher proportions were reported in the present study because it was conducted in a health facility setting with daily outpatient department (OPD) footfall of above 200 at both the PHCs combined, whereas the NNMS was a community-level survey with a larger sample size. Adults attending OPD for regular treatment are motivated and have overall good health-seeking behavior. Studies in Kerala [15] and Africa [16], which were also community-based surveys, reported control rates (control status among those aware) as 36.4% and 8%, respectively. A study conducted in Kerala by Sreelal et al. [17] using prescription data from public health facilities reported a control rate of 31.5% among participants. The proportion was somewhat comparable to the present study because it was conducted in different health facilities.

This study identified determinants significantly associated with control status among adults with hypertension when followed up for six months. Factors identified were sex, SBP, and adequacy of follow-up. When followed for six months, males had 0.5 odds of being controlled in comparison to females. Similar findings were observed in a study conducted by Choi et al. [18] among the Korean population and Cao et al. [15] among the Indian population. It was observed that men tend to have fewer regular follow-up visits in the outpatient department as they were at their respective jobs as compared to women [19]. Adults with high SBP at baseline had lesser chances of being controlled at the end of the six-month follow-up with an odds ratio of 0.5. The possible explanation could be that the six-month period was not long enough to lower SBP among adults with higher baseline SBP [20]. Adequate follow-up was significantly associated with control status among adults with hypertension, with an odds ratio of 2.34. Peng et al. [21] conducted a study among the Chinese population and reported similar results. Regular follow-up will not only increase awareness but also increase adherence and compliance to medication among adults [22-24]. The present study showed that an adult with hypertension needs to be followed up for at least six months to achieve control status. The study also showed that control rates significantly improved when adults with hypertension were followed for three and six months.

The present study reported control rates among adults with diabetes as 24.6%, 28.7%, and 35.9% at baseline, third- and sixth-month follow-up. NNMS 2017-18 reported a control rate of 15.7% among adults [25]. The difference in proportion was due to different sample sizes and methodologies [26]. Adults attending OPD are self-motivated and have overall good health-seeking behavior. Feng et al. [27] conducted a community-based study among the Chinese population and reported control rates of 22.4% and hence reported lower percentages than the present study. China has a more aging population which may further lead to decreased insulin sensitivity and hence poor control rates [28]. A statistically insignificant increase in control rates was also observed among these adults when followed up for three and six months. A possible reason could be that a longer follow-up period was needed to obtain significant results.

Data used in the present study was the one collected under the NPCDCS program. The quality of the data was good as it was filled, updated, maintained, monitored, and supervised by trained health professionals (nurse and auxiliary nurse midwife) and medical officers. Hence the study delivers the use of program data to generate local evidence. It is a first-of-its-kind study reporting control rates and its determinants among adults with hypertension and diabetes above 30 years of age attending the NCD clinic at PHCs from north India. Variables used in the study were in line with the national program. There were no significant differences between the baseline characteristics of adults who didn't come for regular follow-up visits, and hence, it can be generalized to adults attending outpatient health facilities.

The present study has limitations that need to be considered. First, it is a facility-based study, and hence it cannot be extrapolated to the general population. Second, there can be other factors that might affect our findings but are not included in this study, like unhealthy diet, stress, and physical inactivity. An indirect measure of physical activity was used in the form of the BMI of the patient. Third, assumptions were made that all the adults followed up were adherent and compliant with medication. This study reports only associations, not causality, which is a limitation of the study design. Fourth, the health-seeking behavior of the community decides the visit to an NCD clinic, and hence finding of this study will be applicable to populations with similar health-seeking behavior.

Future longitudinal studies are warranted with long-term follow-up so that temporality can be established. Our study findings have observed that almost one-third of adults did not turn up to the NCD clinic for their follow-up. This has to be minimized. The other factors playing a vital role in the development of disease and achieving control status should be deeply studied.

## Conclusions

Control status among adults with hypertension was achieved in slightly more than half of the participants after six months of regular follow-up at the NCD clinic. Similarly, control status among adults with diabetes was achieved in one-third of the participants after six months of regular follow-up at the NCD clinic. Control rates significantly increased among hypertensive patients when followed up for three and six months. Adequate follow-up improved the control status among these patients. Diabetic patients should be followed for more than six months to have significant control status.

Our study showed the use of data collected in programmatic settings for the generation of local scientific evidence. Such data not only strengthen initiatives under the national health program but also help to make public health gains at a community level.
